# Duplication, concerted evolution and purifying selection drive the evolution of mosquito vitellogenin genes

**DOI:** 10.1186/1471-2148-10-142

**Published:** 2010-05-13

**Authors:** Song Chen, Jennifer S Armistead, Katie N Provost-Javier, Joyce M Sakamoto, Jason L Rasgon

**Affiliations:** 1 The W. Harry Feinstone Department of Molecular Microbiology and Immunology, Bloomberg School of Public Health, Johns Hopkins University, Baltimore, MD, 21205, USA; 2 The Institute for Genome Sciences, University of Maryland School of Medicine, Baltimore, MD, 21201, USA; 3 The Johns Hopkins Malaria Research Institute, Bloomberg School of Public Health, Johns Hopkins University, Baltimore, MD, 21205, USA

## Abstract

**Background:**

Mosquito vitellogenin (*Vtg*) genes belong to a small multiple gene family that encodes the major yolk protein precursors required for egg production. Multiple *Vtg *genes have been cloned and characterized from several mosquito species, but their origin and molecular evolution are poorly understood.

**Results:**

Here we used *in silico *and molecular cloning techniques to identify and characterize the evolution of the *Vtg *gene family from the genera *Culex*, *Aedes/Ochlerotatus*, and *Anopheles*. We identified the probable ancestral *Vtg *gene among different mosquito species by its conserved association with a novel gene approximately one kilobase upstream of the start codon. Phylogenetic analysis indicated that the *Vtg *gene family arose by duplication events, but that the pattern of duplication was different in each mosquito genera. Signatures of purifying selection were detected in *Culex*, *Aedes *and *Anopheles*. Gene conversion is a major driver of concerted evolution in *Culex*, while unequal crossover is likely the major driver of concerted evolution in *Anopheles*. In *Aedes*, smaller fragments have undergone gene conversion events.

**Conclusions:**

The study shows concerted evolution and purifying selection shaped the evolution of mosquito *Vtg *genes following gene duplication. Additionally, similar evolutionary patterns were observed in the *Vtg *genes from other invertebrate and vertebrate organisms, suggesting that duplication, concerted evolution and purifying selection may be the major evolutionary forces driving *Vtg *gene evolution across highly divergent taxa.

## Background

Vitellogenin (*Vtg*) genes encode the major yolk protein precursors which are utilized in oviparous organisms to provide nutrition for the developing embryo. In oviparous vertebrates, *Vtg*s are synthesized in the liver of the mature female under the control of estrogen, secreted into the bloodstream, transported to the ovary and selectively taken up by the oocytes [1,2]. In insects, *Vtg*s are synthesized primarily in the fat body of female adults under the regulation of juvenile hormone and/or 20-hydroxyecdysone (20E), secreted into the hemolymph and taken up by the developing oocytes via receptor-mediated endocytosis [3-8].

The female adults of many mosquito species require a vertebrate blood meal to develop eggs, leading to the transmission of a variety of pathogens in humans, wildlife and domestic animals. Understanding the molecular mechanism of blood meal or nutrition-induced synthesis of *Vtg *proteins may lead to insights for novel mosquito control strategies. Great gains have been made in understanding the mechanisms that regulate blood meal-induced vitellogenesis the mosquito *Aedes aegypti*. The cDNA encoding the *Ae. aegypti VgA1 *gene has been characterized and its genomic sequence containing 2015 bp of the 5' promoter region cloned [9,10]. Studies on transcriptional expression and regulation identified a combination of nutritional stimuli (free amino acids) and the steroid hormone 20E as the key factors required for activation of vitellogenesis. The expression of *Vtg*s and other yolk precursor protein (YPP) genes is inhibited by the AaGATAr transcription factor during the previtellogenic period. After digesting a blood meal, amino acids are released from the midgut and activate the TOR signalling pathway in the fat body, resulting in the subsequent de-repression of YPP gene transcription by displacing AaGATAr with another GATA factor [11-16]. Since mosquito *Vtg *synthesis is activated by a blood meal in a sex-, tissue-, and stage-specific manner, its promoter region has been used to control the precise temporal and spatial expression of exogenous genes (such as anti-pathogen effector molecules) in engineered mosquitoes [17-20].

Recently, several new *Vtg *gene sequences were isolated from several mosquito species including *Anopheles albimanus*, *Ae. aegypti*, *Ae. polynesiensis*, *Ae. albopictus*, *Ochlerotatus atropalpus*, *Oc. triseriatus*, *Culex pipiens *and *Toxorhynchites amboinensis *[21]. Comparative sequence analysis performed among three *Vtg *genes from *Ae. aegypti *suggested that *Vg-A1 *and *Vg-B *were closely related and possibly arose by a recent gene duplication event, while *Vg-C *was distantly related to the *Vg-A1 *and *Vg-B *lineage, and possibly arose by an earlier gene duplication event [21]. Nevertheless, the study of the evolution of the *Vtg *gene family among mosquitoes in general is still limited. In this paper, we used *in silico *and molecular cloning techniques to identify and characterize the evolution of the *Vtg *gene family from the genera *Culex*, *Aedes*, *Ochlerotatus *and *Anopheles*. We were also able to identify the probable ancestral *Vtg *gene copy among mosquito genera.

## Results

### Isolation of mosquito *Vtg *genes by *in silico *whole genome analysis and molecular cloning

The release of the whole genome sequences of *Cx. pipiens, Ae. aegypti and An. gambiae *enabled us to analyze the genomic organization of *Vtg *genes and examine their evolutionary pattern across mosquito genera. By using a *Vtg *gene from *Ae. aegypti *(GenBank accession L41842) as query to BLAST search the *Cx. pipiens *whole genome sequence, four intact *Vtg *genes were identified, designated as *CpVg1a *(GenBank accession NZ_AAWU01017720), *CpVg1b *(GenBank accession NZ_AAWU01017726), *CpVg2a *and *CpVg2b *(GenBank accession AAWU01001936) ("*Cp*" refers to the first letters of genus and species name; other mosquito *Vtg *genes in the following text are designated in a similar fashion). Each of these genes contained a different sequence in their 5' promoter regions, indicating they were positioned at different genomic loci. *CpVg2a *and *CpVg2b *were clustered together and organized in a "tail-to-tail" orientation with an intergenic region of 5,113 bp. It was not clear whether *CpVg1a *and *CpVg1b *were clustered due to limitations in the genome assembly. The two *Vtg *families *CpVg1 *and *CpVg2 *shared 64.8%-65.7% nucleotide identity, while subfamily *CpVg1a *and *CpVg1b *shared extremely high nucleotide identity (98.9%), as did *CpVg2a *and *CpVg2b *(98.4%) (Table 1).

**Table 1:** Pairwise nucleotide similarity comparisons of *Culex Vtg *genes^a^.

	*CtVg1a*	*CtVg1b*	*CtVg2a*	*CtVg2b*	*CpVg1a*	*CpVg1b*	*CpVg2a*	*CpVg2b*
**5'end/3'end**								
*CtVg1a*	-	97.6	43.5	45.9	84.3	85.0	44.1	45.3
*CtVg1b*	NM	-	43.2	45.6	83.5	84.3	45.2	45.3
*CtVg2a*	NM	NM	-	94.6	44.4	45.8	73.9	72.5
*CtVg2b*	NM	NM	NM	-	47.3	47.3	74.0	72.7
*CpVg1a*	NM	NM	NM	NM	-	99.2	45.9	44.5
*CpVg1b*	NM	NM	NM	NM	100	-	45.3	44.5
*CpVg2a*	NM	NM	NM	NM	NM	NM	-	96.5
*CpVg2b*	NM	NM	NM	NM	NM	NM	NM	-
**Exon1/Exon2**								
*CtVg1a*	-	100	62.5	62.5	100	100	62.5	62.5
*CtVg1b*	98.1	-	62.5	62.5	100	100	62.5	62.5
*CtVg2a*	65.7	65.8	-	100	62.5	62.5	100	100
*CtVg2b*	65.7	65.7	97.5	-	62.5	62.5	100	100
*CpVg1a*	91.3	91.8	66.5	66.3	-	100	62.5	62.5
*CpVg1b*	91.6	91.9	66.4	66.3	98.7	-	62.5	62.5
*CpVg2a*	65.9	66.0	90.4	91.0	66.8	66.9	-	100
*CpVg2b*	65.8	65.9	90.2	90.9	66.6	66.7	99.6	-
**Intron1/Intron2**								
*CtVg1a*	-	93.8	57.1	57.1	81.5	81.8	53.5	53.5
*CtVg1b*	88.5	-	55.1	56.9	83.1	83.1	54.3	54.3
*CtVg2a*	52.0	54.7	-	93.6	52.7	56.0	75.4	75.4
*CtVg2b*	52.0	54.7	100	-	54.1	54.7	70.8	70.8
*CpVg1a*	66.7	68.8	57.9	57.9	-	90.6	53.4	53.4
*CpVg1b*	69.7	72.7	55.1	55.1	92.3	-	51.4	51.4
*CpVg2a*	50.7	51.3	63.3	63.3	52.0	51.3	-	100
*CpVg2b*	51.3	52.0	64.6	64.6	51.4	51.3	98.7	-
**Exon3/Entire coding region**								
*CtVg1a*	-	98.3	64.6	64.7	92.3	92.2	65.5	65.3
*CtVg1b*	98.1	-	64.9	65.1	92.3	92.2	66.1	65.8
*CtVg2a*	65.5	64.6	-	94.6	63.8	64.0	86.6	85.8
*CtVg2b*	64.3	64.5	97	-	64.0	64.1	84.8	87.8
*CpVg1a*	91.4	91.9	64.8	64.8	-	99.4	65.3	64.6
*CpVg1b*	91.6	91.9	64.8	64.8	98.9	-	65.6	64.9
*CpVg2a*	64.8	65.0	89.7	89.7	65.6	65.7	-	92.9
*CpVg2b*	64.7	64.9	90.3	90.3	65.3	65.4	98.4	-

^a ^Above the diagonal shows percentages of nucleotide identity in 5'end, exon1, intron1 and exon3; below the diagonal shows the percentages of nucleotide identity in the 3'UTR, exon2, intron2 and putative total converted sequences

NM: represents no significant match

BLAST searches revealed three *Vtg *genes in the *Ae. aegypti *genome sequence (*AeVgA*, accession AAGE02018519; *AeVgB*, accession AAGE02009986 and *AeVgC*, accession AAGE02009985), consistent with previous studies [9,10,21]. Based on the genome sequence data, we determined the organization of these three genes. *AeVgB *and *AeVgC *were located in the same super contig (supercont1.191) with an intergenic region of approximately 115 kb (super contig location; *AeVgB*: 160,790-167,359; *AeVgC*: 39,434-45,832), while *AeVgA *was located in a different supercontig (supercont1.477) (VectorBase). These three genes shared 61.4%-88.4% nucleotide identity.

**Figure 1:**
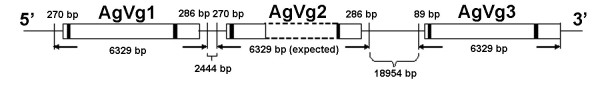
**The organization of *Anopheles gambiae *vitellogenin genes**. The tandem repeat organization was generated by the combination of two available sequences from GenBank (Access numbers AAAB01008880 and AF281078). The thin solid lines represented 5'end and 3'end regions, open boxes represent coding sequences, and the filled boxes represent intron sequences. The dashed lines indicate the unavailable data from whole genome sequence. The sizes of the intergenic regions are indicated by brackets (not to scale). Numbers above the sequences indicate the length in base pairs of the corresponding sequence.

BLAST searches revealed three *Vtg *genes from whole genome sequence of *An. gambiae *(ACCESSION AAAB01008880) and non-redundant database in GenBank (ACCESSION AF281078). The former encoded three *Vtg *genes (*AgVg1, AgVg2 *and *AgVg3*), but with partial coding sequence for *AgVg1 *and *AgVg2*; the later encoded *AgVg1 *and *AgVg2 *with partial sequence for *AgVg2*. The *AgVg2 *sequence was partial due to a genome sequence gap, but by combining data from both datasets we obtained intact sequences for *AgVg1 *and *AgVg3 *(Figure 1). All three *Vtg *genes showed extremely high nucleotide identity (98.1%-99.2%) and are encoded as a tandem repeat in the *An. gambiae *genome (Figure 1).

When we attempted to isolate and clone the 5' promoter region of the *Cx. tarsalis Vtg *gene, the obtained sequence data lead us to identify four different loci that had high conservation in the coding sequence but unique 5'-promoter regions. On the basis of the *Cx. pipiens Vtg *gene coding sequence, multiple sets of primers were designed to isolate the intact *Vtg *genes from *Cx. tarsalis*. We first cloned the partial coding sequences and then obtained the 5' and 3' end fragments. Specific primers located in the 5' promoter region and 3'coding or 3'UTR regions were designed to amplify the full length genes. Four full length *Vtg *genes from *Cx. tarsalis *were isolated, designated as *CtVg1a*, *CtVg1b*, *CtVg2a *and *CtVg2b*. Sequence analysis indicated that the two *Vtg *families *CpVg1 *and *CpVg2 *shared 64.3%-65.5% nucleotide identity, while subfamily *CtVg1a *and *CtVg1b *shared extremely high nucleotide identity (98.1%), as did *CtVg2a *and *CtVg2b *(97.0%) (Table 1).

### Identification of the ancestral *Vtg *gene copy in mosquitoes

**Figure 2:**
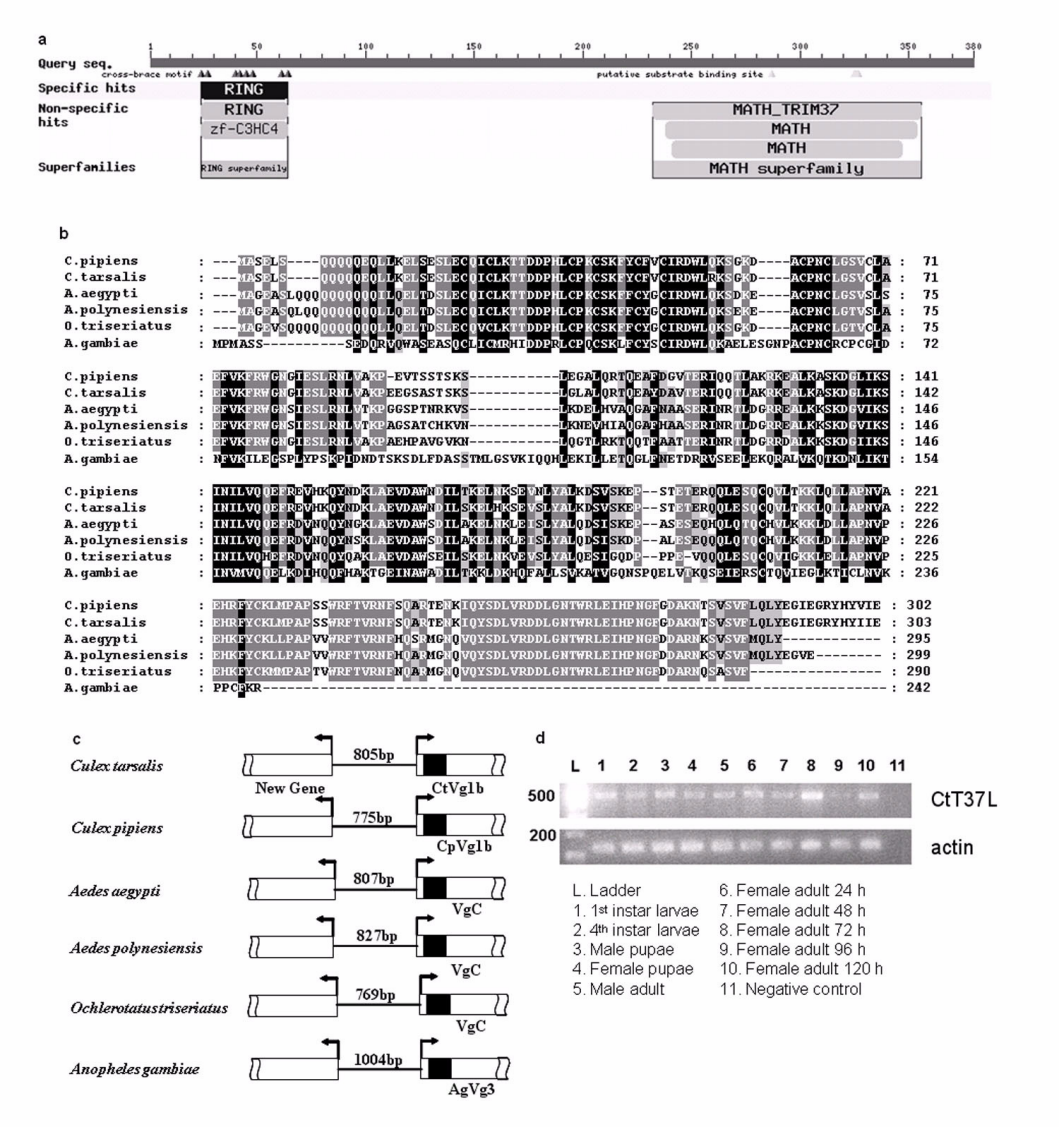
**The identified novel gene T37L in mosquitoes**. (a) T37L protein contains two putative functional protein-protein binding domains: RING-finger domain and MATH domain. (b). Partial amino acid alignment of T37L genes in six mosquito species. (c) Conservative "tail-to-tail" organization between the T37L and a *Vtg *gene in mosquitoes. The open boxes represent coding sequence and the filled box represents intron sequence. The intergenic region sizes are indicated. (d) Transcriptional expression of T37L in *Culex tarsalis*. Accession numbers are: *Culex pipiens*, NZ_AAWU01017726; *Aedes aegypti*, AY373377; *Aedes polynesiensis*, AY691320; *Ochlerotatus triseriatus*, AY691323; *Anopheles gambiae*, AAAB01008880.

While isolating the 5'-promoter region of the *CtVg1b *gene, we identified a novel gene 805 bp upstream of the *CtVg1b *start codon, in the opposite orientation. This small gap between the start codons of the novel gene and *CtVg1b *suggests that this region may act as a bidirectional promoter. The novel gene contained two putatively functional protein-protein binding domains - a RING-finger domain and MATH domain (Figure 2a) and has homology to the human tripartite motif protein 37 (TRIM37) gene - we therefore refer to this gene as TRIM37-like (T37L) protein. By examining *Vtg *gene sequence data from other mosquitoes (*Cx. pipiens, Ae. aegypti, Ae. polynesiensis, An. gambiae and Oc. triseriatus*) we identified T37L homologues upstream of one of the *Vtg *copies in all species examined (Figure 2). Among mosquitoes, the size of the intergenic region ranged from 769 bp to 1004 bp (Figure 2). The T37L amino acid sequences were highly conserved among mosquito species (Figure 2). RT-PCR showed that in *Cx. tarsalis CtT37L *was transcriptionally expressed in all developmental stages (Figure 2). Interestingly, the down-stream associated *Vtg *gene (*CtVg1b*) is also expressed in all developmental stages [22]. The discovery of conservation of the T37L-*Vtg *structure in all examined mosquito genomes indicated the *Vtg *gene in this special organization was likely the ancestral origin of mosquito *Vtg *genes through gene duplication events.

### Identification of orthologues and paralogues between closely-related mosquito species

**Figure 3:**
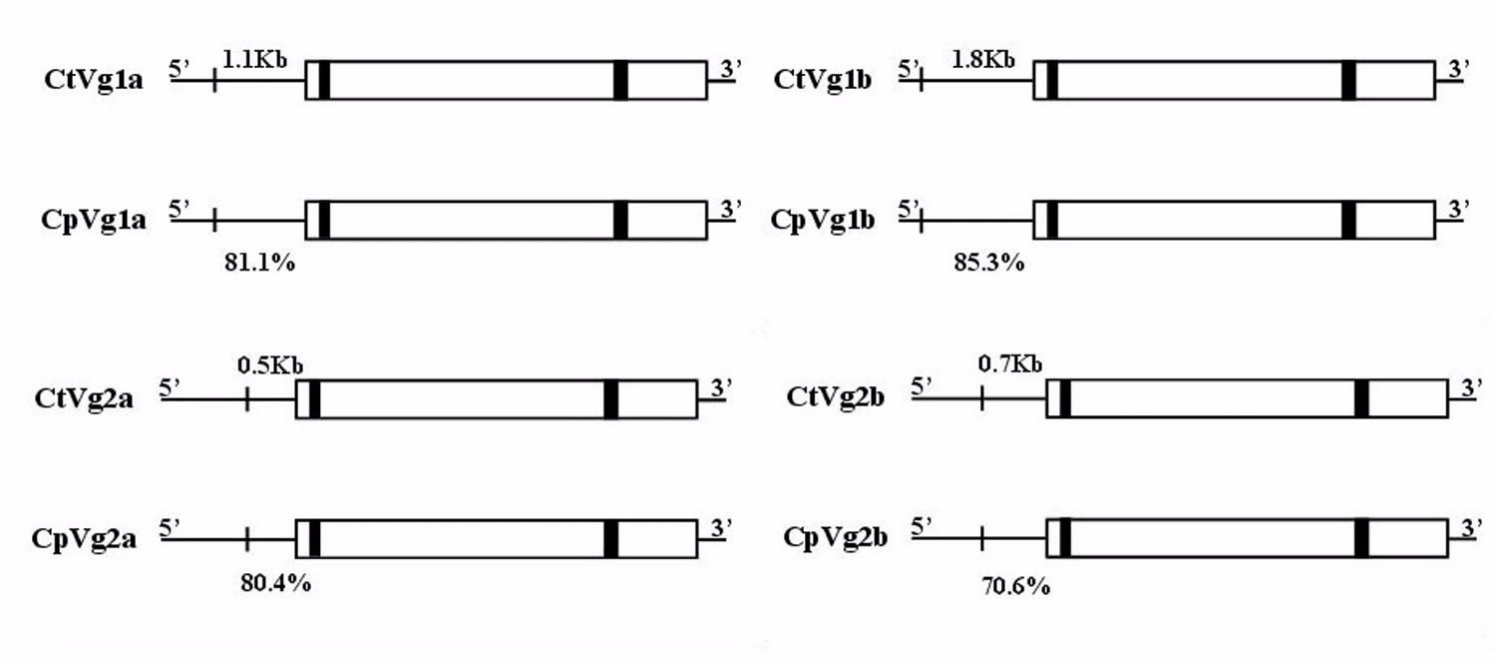
**Orthologous/paralogous relationship of *Vtg *genes in *Cx. tarsalis *and *Cx pipiens***. Orthologous and paralogous gene relationships were determined by nucleotide identity of their 5'promoter regions. The thin solid lines represent 5'promoter and 3'end regions, open boxes represent coding sequences, and the filled box represents intron sequence. Sequence direction (5' and 3') and the number of nucleotides with high identity between orthologs in their 5' promoter region are indicated above the sequences; the percentage of nucleotide identity is listed below the sequence.

To determine the orthologous relationship of the *Vtg *genes from *Cx. pipiens *and *Cx. tarsalis*, we used their 5' promoter sequences to calculate identity by BLAST analysis (Table 1). The vertical and horizontal directions in Figure 3 show orthologous and paralogous relationship, respectively. The 5' promoter region of each orthologous gene shared 70.6-85.3% nucleotide identity, while producing no significant match with any other *Vtg *gene (Figure 3). For example, *CtVg1a *shared 81.1% nucleotide identity with *CpVg1a *in their 1.1 Kb 5' promoter region directly upstream of the start codon, but no significant match alignment with that region from the other *Vtg *genes.

### Phylogenetic analysis

**Figure 4:**
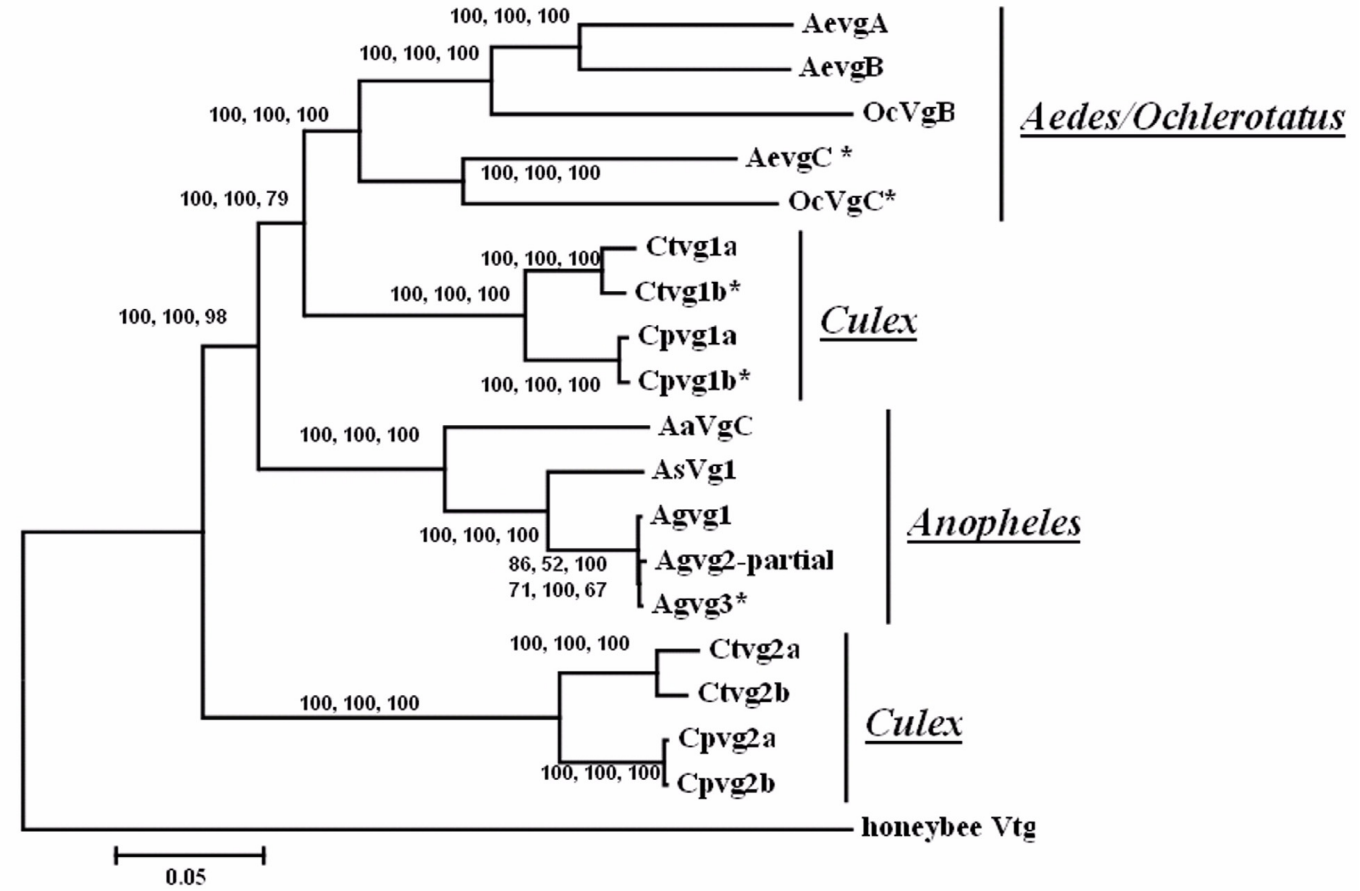
**Phylogenetic analysis of mosquito *Vtg *genes**. Phylogenetic analysis of mosquito vitellogenin gene sequences was conducted using Maximum Likelihood, Bayesian and Neighbor-joining methods; numbers at nodes indicate support values for each method (in that order). T37L-associated (putative ancestral) *Vtg *copies in different mosquito species are indicated by "*". *Vtg *gene of honey bee *Apis mellifera *(accession number AJ517411) was used as outgroup. *Vtg *gene accession numbers are AJ517411 for honey bee, AY691327 for *AaVgC *and the remainder are listed in Table 2.

Phylogenetic analysis using the full *Vtg *coding sequences was used to examine the evolution of *Vtg *genes among mosquitoes (Figure 4). All analyses gave identical tree morphology. With the exception of the *Culex Vg2 *group (which represents a duplication event unique to the genus *Culex*), all other mosquito *Vtg *genes conformed to three major clades: *Aedes/Ochlerotatus*, *Culex *and *Anopheles*. The *Aedes/Ochlerotatus *clade is more closely related to the *Culex Vg1 *clade than the *Anopheles *clade, which is in agreement with the agreed relationships of these genera. Paralogous copies cluster more closely than orthologues within each clade. Three independent duplication patterns were observed among mosquito genera. In *Culex*, a divergent duplication event produced the *Vg1 *and *Vg2 *genes, which each then underwent additional independent duplications generating four *Vtg *genes in total. These duplication events occurred prior to the divergence of *pipiens *and *tarsalis *within the genus. The absence of *Vg2 *homologues in non-*Culex *genera may reflect a unique duplication event in *Culex*, or less likely, independent gene loss events in *Anopheles *and *Aedes*/*Ochlerotatus*. In *Aedes*, the early duplication from the ancestral copy generated the *VgA*/*B *lineage, which underwent an additional duplication to generate 3 *Vtg *copies - these duplication events occurred prior to the divergence of the *Aedes *and *Ochlerotatus *genera. In *Anopheles*, *Vtg *gene duplication events occurred rapidly during tandem repeat generation (Figure 4). Unlike *Culex *and *Aedes*/*Ochlerotatus*, none of the *An. gambiae Vtg *genes cluster with other members of the genus, suggesting that the rapid tandem repeat generation occurred after the split of *gambiae *from the other members of the *Anopheles *genus.

### Concerted evolution between mosquito *Culex *gene paralogues *Vg1a *and *Vg1b*

Concerted evolution occurs in multigene families and is expected to generate a higher sequence similarity between paralogues than between orthologues. Statistically significant fragments were identified in paralogues *Vg1a *and *Vg1b *in the genus *Culex*. The predicted conversion fragments include not only coding regions but also non coding DNA such as 5' promoter regions and introns (Table 2).

**Table 2:** Detection of gene conversion events in mosquito vitellogenin genes by GENECONV^#^, showing the length and location of the converted genomic regions.

Mosquito	Gene1	Gene2	p-value*	Conversion tract**			Entire gene size[bp]***
				Begin	End	Length [bp]	
*Culex tarsalis*	*CtVg1a*	*CtVg1b*	0.0000	-101(-101)	6479(6484)	6580(6585)	6478(6483)
	*CtVg2a*	*CtVg2b*	0.0000	-161(-160)	6208(6223)	6369(6383)	6380(6395)
*Culex pipiens*	*CpVg1a*	*CpVg1b*	0.0000	-123(-123)	6480(6468)	6603(6591)	6476(6464)
	*CpVg2a*	*CpVg2b*	0.0000	-167(-170)	6103(6108)	6270(6278)	6434(6433)
*Anopheles gambiae*	*AgVg1*	*AgVg3*	0.0000	-41(-41)	6055(6055)	6096(6096)	6329(6329)
	*AgVg1*	*AgVg2*	0.0000	-235(-235)	1421(1421)	1656(1656)	6328(1421)
	*AgVg2*	*AgVg3*	0.0000	-88(-88)	1421(1421)	1509(1509)	1421(6329)
							
*Anopheles stephensi*	*AsVg1*	*AsVg2*	0.0000	-325(-325)	190(190)	515(515)	6380(190)
*Aedes aegypti*	*AeVgA*	*AeVgB*	0.0000	4226(4223)	4867(4864)	642(642)	6574(6570)
	*AeVgA*	*AeVgB*	0.0000	5397(5396)	5877(5876)	481(481)	6574(6570)
	*AeVgB*	*AeVgC*	0.0478	6196(6016)	6218(6038)	23(23)	6570(6399)
*Ochlerotatus atropalpus*	*OcVgB*	*OcVgC*	0.0000	1373(1226)	1595(1448)	223(223)	6459(5624)
	*OcVgB*	*OcVgC*	0.0050	1700(1571)	1830(1701)	131(131)	6459(5624)
	*OcVgB*	*OcVgC*	0.0050	3678(3549)	3772(3643)	95(95)	6459(5624)
	*OcVgB*	*OcVgC*	0.0229	3885(3756)	3961(3832)	77(77)	6459(5624)

*p-value is Sim-p value. For *AgVg2 *and *AsVg2 *only partial sequences were available

** location: using "A" in start codon as "+1"; numbers in brackets represent the location or size of Gene2

***exons+introns

#Accession numbers for mosquito *Vtg *genes are: CpVg1a, NZ_AAWU01017720; CpVg1b, NZ_AAWU01017726; CpVg2a and CpVg2b, AAWU01001936; AgVg1 and AgVg2, AF281078;AgVg3, AAAB01008880; AsVg1 and AsVg2, DQ442990; AeVgA, L41842; AeVgB, AY380797; AeVgC, AY373377; OcVgB, AY691321; OcVgC, AY691322.

Though concerted evolution certainly plays an important role in maintaining the high level of mosquito *Vtg *gene sequence conservation, an alternative explanation for high sequence homogeneity is "birth and death" evolution under strong purifying selection [23-25]]. The two models of evolution could be distinguished by comparing the number of synonymous differences per site (Ps) within and between species. In the presence of strong purifying selection without concerted evolution, DNA sequence differences will be observed primarily at the synonymous sites. If a gene has undergone concerted evolution, sequence differences will not be biased toward synonymous sites. If strong purifying selection is responsible for the observed pattern, intraspecific and interspecific synonymous differences are expected to be similar. If intraspecific synonymous differences are much lower than interspecific difference, this would suggest that concerted evolution is the dominant force. In our dataset, Ps values in some paralogous pairs are much lower than that in orthologous pairs. For example, Ps values ranged from 0.0316 to 0.0525 in the paralogous pairs *CtVg1a*-*CtVg1b *and *CpVg1a*-*CpVg1b*, but ranged from 0.1809 to 0.1851 in the orthologous combinations (Table 3). The Ps value range of 0.0316 to 0.0525 was far from saturation level (0.4-0.7 in many species) [23]. Moreover, extremely high sequence conservation was observed between paralogous introns (88.5% to 100%), but was lower between orthologous introns (63.3% to 81.5%) in *Culex *(Table 1) which may not be explained simply by purifying selection. Taken together, the data demonstrated that strong purifying selection is likely not responsible for high sequence identity between gene paralogues within the same family.

**Table 3:** Numbers of synonymous P_S _(SE) and nonsynonymous P_N _(SE) differences per site in mosquito *Vtg *genes.

	Gene1	Gene2	P_S_(SE)	P_N_(SE)
Orthologues	*Ctvg1a*	*Cpvg1a*	0.1851(0.0097)	0.0457(0.0036)
	*CtVg1b*	*CpVg1b*	0.1809(0.0093)	0.0432(0.0034)
	*Ctvg2a*	*Cpvg2a*	0.2377(0.0102)	0.0514(0.0038)
	*Ctvg2b*	*Cpvg2b*	0.2277(0.0105)	0.0513(0.0039)
	*AgVg1*	*AsVg1*	0.2015(0.0092)	0.0276(0.0029)
	*AgVg3*	*AsVg1*	0.2020(0.0095)	0.0302(0.0031)
	*AeVgB*	*OcVgB*	0.4881(0.0134)	0.1208(0.0062)
	*AeVgC*	*OcVgC*	0.4872(0.0142)	0.2535(0.0093)

Paralogues				
*Culex tarsalis*	*CtVg1a*	*CtVg1b*	0.0525(0.0054)	0.0047(0.0011)
	*CtVg1a*	*CtVg2a*	0.4941(0.0114)	0.3246(0.0088)
	*CtVg1a*	*CtVg2b*	0.4936(0.0116)	0.3232(0.0089)
	*CtVg1b*	*CtVg2a*	0.4909(0.0117)	0.3226(0.0087)
	*CtVg1b*	*CtVg2b*	0.4930(0.0120)	0.3216(0.0088)
	*CtVg2a*	*CtVg2b*	0.0564(0.0052)	0.0136(0.0019)
*Culex pipiens*	*CpVg1a*	*CpVg1b*	0.0316(0.0039)	0.0033(0.0010)
	*CpVg1a*	*CpVg2a*	0.4708(0.0116)	0.3233(0.0093)
	*CpVg1a*	*CpVg2b*	0.4689(0.0120)	0.3236(0.0094)
	*CpVg1b*	*CpVg2a*	0.4658(0.0119)	0.3231(0.0094)
	*CpVg1b*	*CpVg2b*	0.4640(0.0122)	0.3234(0.0095)
	*CpVg2a*	*CpVg2b*	0.0334(0.0037)	0.0087(0.0017)
Anopheles gambiae*	*AgVg1*	*AgVg3*	0.0226(0.0031)	0.0072(0.0013)
Aedes aegypti**	*AeVgA*	*AeVgB*	0.3294(0.0118)	0.0663(0.0045)
	*AeVgA*	*AeVgC*	0.4758(0.0119)	0.2488(0.0072)
	*AeVgB*	*AeVgC*	0.4839(0.0126)	0.2461(0.0075)
*Ochlerotatus atropalpus*	*OcVgB*	*OcVgC*	0.5283(0.0121)	0.2563(0.0087)

*AgVg2 not used for calculation due to partial available sequence

**Ps value for *AeVgA*-*AeVgB *two conversion tracts (648 bp, 496 bp) are 0.1958(0.0286) and 0.0283 (0.0137)

### Purifying selection between families *Vg1 *and *Vg2 *in genus *Culex*

To examine the selection pressure between the two paralogous families *Vg1 *and *Vg2 *in *Culex *species, dN/dS values were calculated between *Vg1 *and *Vg2*. All pairwise comparisons were significantly less than 1 (p < 0.0001, Z-test), indicating that these *Vtg *sequences are under purifying selection (Table 4). No significant conversion tracts were detected using GENECONV (data not shown). To test if positive selection was acting on different regions of mosquito *Vtg *genes, we used WSPMaker http://wspmaker.kobic.kr/, a web tool for scanning and calculating selection pressures in sub-regions of two protein-coding DNA sequences [26]. No dN/dS ratio was significantly greater than 1 (data not shown).

**Table 4:** dN/dS ratios and evidence for purifying selection between two paralogous families *Vg1 *and *Vg2 *in *Culex*.

mosquito	*Vg1*	*Vg2*	dN/dS	p-value (Z-test)
*Culex pipiens*	*CpVg1a*	*CpVg2a*	0.5726	0.0000

	*CpVg1a*	*CpVg2b*	0.5792	0.0000

	*CpVg1b*	*CpVg2a*	0.5824	0.0000

	*CpVg1b*	*CpVg2b*	0.5890	0.0000

*Culex tarsalis*	*CtVg1a*	*CtVg2a*	0.5300	0.0000

	*CtVg1a*	*CtVg2b*	0.5279	0.0000

	*CtVg1b*	*CtVg2a*	0.5301	0.0000

	*CtVg1b*	*CtVg2b*	0.5246	0.0000

### Mosaic evolution between paralogues *Vg2a *and *Vg2b in Culex*, and paralogues in *An. gambiae*, *Ae. aegypti *and *Oc. atropalpus*

We observed that gene conversion events were variable in the amount of gene sequence involved between mosquito species. While in some species, almost the entire gene sequences have undergone concerted evolution, others had a mosaic pattern of conversion events, where some segments of the genes are homogenized and evolved in concert, while others diverged without gene conversion [27]. We determined that sequence homogenization by gene conversion occurred in the entire gene sequences of *Culex Vg1 *paralogues, while homogenization occurred only partially in *Culex Vg2 *paralogues, *An. gambiae *paralogues, *Ae. aegypti *paralogues and *Oc. atropalpus *paralogues (Table 2). The lower number of synonymous differences per site (Ps) found in paralogues of *CpVg2a*-*CpVg2b*, *CtVg2a*-*CtVg2b *and *AgVg1*-*AgVg3 *(ranging from 0.0226-0.0564), suggests that sequence homogenization was not due to strong purifying selection (Table 3). We analyzed dN/dS ratios between the unconverted coding region of the paralogues *Vg2a *and *Vg2b *in *Culex*, and paralogues in *An. gambiae*, *Ae. aegypti *and *Oc. atropalpus*. All unconverted coding regions have likely undergone purifying selection (dN/dS < 1, p < 0.01, Z-test) (Table 5).

**Table 5:** Purifying selection on unconverted coding region of mosquito *Vtg *genes.

mosquito	Gene1	Gene2	Size of non-converted region[bp]†	P_S_	dN	dS	dN/dS	p-value (Z-test)
*Culex pipiens*	*CpVg2a*	*CpVg2b*	331(331)	0.5314	0.1772	0.9246	0.1917	0.0003

*Culex tarsalis*	*CtVg2a*	*CpVg2b*	172(172)	0.5887	0.3456	1.1524	0.2999	0.0191

*Anopheles gambiae*	*AgVg1*	*AgVg3*	274(274)	0.3868	0.1344	0.5439	0.2471	0.0002
*Aedes aegypti*								
Fragment1	*AeVgA*	*AeVgB*	4329(4314)	0.3548	0.0706	0.4805	0.1469	0.0000
Fragment2	*AeVgA*	*AeVgB*	472(472)	0.2920	0.1053	0.3700	0.2846	0.0001
Fragment3	*AeVgA*	*AeVgB*	697(694)	0.3852	0.1109	0.5405	0.2052	0.0000
Fragment1	*AeVgB*	*AeVgC*	6055(5881)	0.4960	0.2876	0.8121	0.3454	0.0000
Fragment2	*AeVgB*	*AeVgC*	352(361)	0.3089	0.4010	0.3981	1.0073	1.0000*

Fragment1-2 (combined)	*AeVgB*	*AeVgC*		0.4870	0.2942	0.7860	0.3743	0.0000
*Ochlerotatus atropalpus*								
Fragment1	*OcVgB*	*OcVgC*	1298(1145)	0.5889	0.2581	1.1536	0.2237	0.0000
Fragment2	*OcVgB*	*OcVgC*	104(123)	0.6291	0.4917	1.3687	0.3592	0.0508*
Fragment3	*OcVgB*	*OcVgC*	1847(1847)	0.5320	0.2651	0.9267	0.2861	0.0000
Fragment4	*OcVgB*	*OcVgC*	112(112)	0.6267	0.3979	1.3541	0.2938	0.0569*
Fragment5	*OcVgB*	*OcVgC*	2437(1724)	0.4952	0.4549	0.8096	0.5619	0.0000

Fragment1-5 (combined)	*OcVgB*	*OcVgC*		0.5369	0.3306	0.9436	0.3504	0.0000

*no significant selection pressure on these fragments

†Size Gene 1(Size Gene 2)

## Discussion

T37L was accidentally identified during attempts to isolate the 5-prime regulatory region of the *Cx. tarsalis Vg1b *gene. We found that T37L was constitutively expressed throughout mosquito immature and adult development. Interestingly, of the four *Vtg *genes in *Cx. tarsalis*, *Vg1b *is also constitutively expressed throughout immature and mature development, mirroring the expression of T37L [22]. The fact that this gene arrangement (T37L-short regulatory region-*Vtg*) is conserved among mosquitoes suggests that their functions may be linked in some way. Experiments are currently ongoing to investigate the potential functional link between T37L and *Vtg *expression, possibly mediated by the shared putatively bi-directional promoter.

GENECONV is a computational program for statistically detecting high-scoring aligned pairs in a given alignment [28]. It potentially detects both gene conversion and unequal recombination events (two mechanisms to generate concerted evolution) but can not distinguish between the two phenomena. Gene conversion is an event in DNA genetic recombination, where all or part of one gene is converted to the sequence of a nearby homologous gene in a nonreciprocal transfer of genetic information [29]. Gene conversion is used to explain concerted evolution between duplicated genes (two copies) without changing copy number [30,31]. Unequal crossover is a recombination event between misaligned non-allelic sequences on a pair of homologous chromosomes and usually causes copy-number fluctuation by deletion or duplication of the gene region [29]. It is often used to explain concerted evolution within tandemly-arrayed gene families (three or more copies) and always generates duplicates in the same orientation ("head-to-tail"). There is no tandem repeat of *Vtg *genes found in the genomes of genus *Culex*, *Aedes *or *Ochlerotatus*, suggesting that gene conversion rather than unequal crossover generated the concerted evolution of *Vtg *genes in these mosquito species. In contrast, in *An. gambiae *unequal crossover may contribute the concerted evolution of *Vtg *genes due to the tandem repeat organization ("head-to-tail") of the *Vtg *genes that share high sequence conservation. Different numbers of *Vtg *tandem copies have been described between different mosquito strains (*An. gambiae *G3 strain: 3 copies vs. PEST strain: 7 copies [32], suggesting unequal crossover may generate *Vtg *copy number fluctuations in *Anopheles*.

In this study, we determined that after arising by duplication events, purifying selection and concerted evolution drive the evolution of mosquito *Vtg *genes. Similar patterns have been observed in the *Vtg *genes of other invertebrate and vertebrate organisms. Among the six *Vtg *genes (*Vit-1 *to *Vit-6*) of the nematode *Caenorhabditis elegans*, high sequence identity was observed between *Vit-1 *and *Vit-2 *(95% nt identity) and among *Vit-3*, *Vit-4 *and *Vit-5 *(above 96%), while *Vit-3 *and *Vit-4 *are located in tandem on the X chromosome and are nearly identical to each other in the coding region [33]. Two *Vtg *genes located at different loci were identified from the cockroach *Leucophaea maderae *which shared high similarity (96% identity at the amino acids level) [34]. In vertebrates, four *Vtg *genes have been identified from the frog *Xenopus laevis *which fall into two pairs that share approximately 95% sequence identity within pairs, while approximately 65.5% identity between pairs [1]. A genomic region harboring two *Vtg *genes that shared almost 99% sequence identity and that were separated from each other by a 4.5-kb intergenic region was isolated from the rainbow trout *Oncorhynchus mykiss *[35]. *O. mykiss *also contains a locus containing twenty complete *Vtg *genes and ten pseudogenes per haploid genome that show a high degree of similarity at the sequence level (97.4%-100%), although these genes differ from each other by retrotransposon-like sequence insertions, deletions and rearrangement events [36]. Other salmonid fishes show a similar pattern [37]. Gene conversion has been suggested as one of the forces driving evolution of some fish *Vtg *genes [38], but has not been systematically examined across diverse taxa. Re-analysis of sequence data from vertebrates and invertebrates show that concerted evolution and/or purifying selection may be a general property of *Vtg *gene evolution across highly divergent taxa [additional file 1, 2]. However, other evolutionary forces can also drive the evolution of *Vtg *genes. For example, a tandemly-arrayed *Vtg *gene cluster (VGC) has been selectively conserved in most oviparous vertebrate lineages [39,40].

Although both purifying selection and concerted evolution play important roles in maintaining high levels of sequence conservation, they act by different methods. A gene under purifying selection can maintain sequence conservation in protein coding or regulatory regions due to the functional constraint of selection pressure against deleterious variants. Concerted evolution is a molecular process that leads to homogenization of DNA sequences of duplicated regions within a genome. In yeast, it has been shown that the level of concerted evolution is positively correlated with gene expression levels, suggesting that dosage-sensitive genes are likely to favor concerted evolution [41]. *Vtg *is an essential component needed for embryonic development. Increased quantities of egg yolk may have dramatically beneficial effects on embryo survival. The high identity of *Vtg *genes maintained by concerted evolution may be beneficial for mosquito reproduction due to increased expression of this vital yolk protein.

After duplication, the duplicated gene copy can have different fates, such as loss of function through pseudogene formation or diversification of gene function through neofunctionalization [42,43]. It has been reported that *Vtg *gene *vit-1 *in *C. elegans *and about ten *Vtg *genes in rainbow trout were actually pseudogenes [33,36]. In *Cx. tarsalis*, all of four *Vtg *genes are transcriptionally expressed and are not pseudogenes [22]. *Vtg *genes may also gain novel functions through alteration of their transcriptional expression pattern instead of sequence divergence. In the honeybee, *Vtg *is expressed in immature, worker and male castes and is associated with a variety of social behaviours [44-46]. *Vtg *proteins also have important antioxidant activities and prolong lifespan in honey bee and *C. elegans *[47,48]. *Vtg *is also involved in promoting macrophage phagocytosis in fish [49]. In *Cx. tarsalis*, *CtVg1b *is expressed not only in the adult female but is constitutively expressed during larval and pupal developmental stages, while *CtVg1a*, *CtVg2a *and *CtVg2b *are expressed exclusively in the adult stage[22] - it remains to be seen if *CtVg1b *has any alternative function in *Cx. tarsalis *and whether constitutive expression is related to the associated *CtT37L *gene.

## Conclusions

After duplication, two major forces drive the evolution of the vitellogenin (*Vtg*) gene family in mosquitoes. We identified the putative ancestral *Vtg *gene among different mosquito species by its conserved association with a novel gene (T37L) approximately one kilobase upstream of the start codon. Phylogenetic analysis indicated that the *Vtg *gene family arose by unique duplication events in each mosquito genera. Signatures of purifying selection were detected in *Culex*, *Aedes *and *Anopheles*. Gene conversion is a major driver of concerted evolution in *Culex*, while unequal crossover is likely the major driver of concerted evolution in *Anopheles*. In *Aedes*, smaller fragments have undergone gene conversion events. These data, plus reanalysis of *Vtg *gene sequences from other organisms suggest that duplication, concerted evolution and purifying selection may be major evolutionary forces driving *Vtg *gene evolution across highly divergent taxa.

## Methods

### Mosquitoes

*Culex tarsalis *were from the KNWR strain, which was collected in the Kern National Wildlife Refuge (KNWR) in Kern County, California. Larvae were reared at a standard density of 200 larvae/pan and fed a 1:2:2 blend of fish food, rabbit pellets and bovine liver extract. Adults were maintained on 10% sucrose. Mosquitoes were maintained by autogenous oviposition.

### Genomic DNA extraction, total RNA extraction and first strand cDNA synthesis

Genomic DNA was isolated from *Cx. tarsalis *adults using the procedure described in Chen and Li [50]. Total RNA was extracted from female adults using the SV Total RNA Isolation System Kit (Promega) as described by the manufacturer. Two micrograms of RNA was used as template for first strand cDNA synthesis to make 3'RACE-ready cDNA using The SMART RACE cDNA Amplification Kit according to the manufacturer's protocol (Clonetech).

### Cloning full length vitellogenin genes from *Culex tarsalis*

To isolate the full *Vtg *coding sequences, we began by cloning partial coding sequences. Based on the available *An. gambiae*, *Ae. aegypti and Cx. pipiens *vitellogenin sequences, two primers (CpVgCoF1: 5'-CCA-AGA-CCA-TGA-CCG-CCC-TG-3' and CpVgCoR1: 5'-TTT-CCC-ATT-TGG-TTG-GTG-TTG-GG-3') were designed to PCR-amplify the coding sequence near the 5' end. The PCR protocol was 2 min at 94°C, followed by 35 cycles consisting of 94°C for 30 s, 50°C for 30 s, and 72°C for 1 min, and a final 72°C extension for 10 min. PCR products were separated on a 1% agarose gel in 1 × TAE buffer. PCR fragments were cloned into the TOPO TA cloning vector (Invitrogen) and sequenced. Two partial vitellogenin gene (*Vg1 *and *Vg2*) sequences were identified.

The 5'-flanking sequences were obtained using the GenomeWalker Universal Kit (Clonetech) according to the manufacturer's protocol. Briefly, genomic DNA was digested by eight blunt-end restriction enzymes (SnaBI, MscI, SmaI, ScaI, EcorV, Dra, PvuII, Stu I), and then adaptor-ligated on both ends. Use adaptor and gene-specific primers, we amplified the 5' flanking regions. Two gene-specific primers were designed for each gene. Vg1: Vg1GSP1 (5'-CTC-CCT-CCA-GAT-GTT-GGT-ACG-GTA-TCC-3') and Vg1GSP2 (5'-TCG-TTG-AAG-TTG-GCG-TAC-TCG-GCT-TGC-3'); Vg2: Vg2GSP1 (5'-AAG-TCC-GTG-CGG-TGA-CCA-TCG-TCC-AGA-3'); Vg2GSP2 (5'-ATA-CTG-GTT-GAA-CTG-AGC-GTA-TTG-AGC-3'). The nested PCR protocol was 2 min at 94°C, followed by 20 cycles consisting of 94°C for 30 s, 67°C for 4 min, and a final 72°C extension for 10 min; the second PCR reaction was identical except that cycles were increased to 30 and used 1 μl 50× dilution of the first PCR product as template. A 10:1 mix of Taq and pfu DNA polymerase was used for all reactions. PCR fragments were TOPO cloned and sequenced as described above.

3'Rapid Amplification of cDNA ends (3'RACE) approach was used to isolate the 3' end of the *Vtg *genes. Two forward gene-specific primers were designed for each *Vtg *gene for nested 3'RACE PCR on the basis of 3' coding sequence of the *Cx. pipiens Vtg *genes. Vg1: CpVg13F1 (5'-ACT-TCC-AGA-ACG-CTG-ACA-CC-3') and CpVg13F2 (5'-TCA-AGA-ACG-GAT-TCA-GCG-AG-3'); Vg2: CpVg23F1 (5'-GAG-AAC-AAC-CAG-CAG-CAC-CT-3') and CpVg23F2 (5'-ATT-GTG-CCG-AAC-GGT-GCT-CA-3'). The nested PCR protocol was 2 min at 94°C, followed by 20 cycles consisting of 94°C for 30 s, 52°C for 30 s and 72°C for 90 s, and a final 72°C extension for 10 min; the second PCR reaction was identical except that cycles were increased to 30 and used 1 μl 50× diluted PCR product as template. PCR fragments were TOPO cloned and sequenced as described above.

Finally, we designed four pairs of gene-specific primers to amplify the four full length *Vtg *genes, in which forward primers and reverse primers were located in 5' promoter region and 3'UTR region, respectively. Vg1a: Vg1aPF (5'-GAA-CCC-ACC-GAT-TGT-TTA-CG-3') and Vg1aSPR (5'-ATG-CCT-TTG-TAA-ACA-GTT-CC-3'); Vg1b: Vg1bPF (5'-TGG-TGT-TGC-TCT-CAG-ACT-TG-3') and Vg1bSPR (5'-CCA-AAT-TCA-TTG-CTT-TCC-GA-3'); - Vg2a: Vg2aPF (5'-TCA-ACA-ACC-ATC-CCT-TCA-CA-3') and Vg2aSPR (5'-TCC-GTT-ACA-ACC-ATC-TAG-AG-3'); Vg2b: Vg2bPF (5'-AAA-GCA-GCC-TCA-AGC-AAT-CG-3') and Vg2bSPR (5'-GGC-AGT-TGT-ATC-TTC-CAA-GG-3'). The PCR protocol was 2 min at 94°C, followed by 35 cycles consisting of 94°C for 30 s, 50°C for 30 s, and 72°C for 4 min 30 sec, and a final 72°C extension for 10 min. PCR products were cloned and sequenced as described above. The four new isolated *Vtg *genes from *Cx. tarsalis *have been deposited in GenBank under accession numbers GU017909-GU017912.

### Temporal expression of the TRIM37-like protein (CtT37L) gene in *Cx. tarsalis*

While isolating the 5'-promoter region of the *CtVg1b *gene, we identified a novel gene (*CtT37L *- see results) 805 bp upstream of the *CtVg1b *start codon, in the opposite orientation. We determined the expression profile of *CtT37L *in *Cx. tarsalis *by reverse transcriptase PCR. Total RNA was isolated using RNAqueous micro-kit columns (Ambion) from first-instar larvae, fourth instar larvae, pupae (male and female), 24-hour old male adults, and female adults 24, 48, 72, 96 and 120 hours post-emergence. The experiment was replicated 3 times. RNA was extracted from 5 pooled individuals except in the case of first instar larvae where 10 individuals were pooled. Isolated RNA was treated with DNase (Ambion) to eliminate residual DNA contamination. cDNA was generated using 1 μg of DNase-treated RNA for reverse transcription using the Superscript RT for PCR kit (Invitrogen) in 20 μl volumes with Oligo(dT)20 primers. No-RT controls were run for each sample. For RT-PCR 2 μl of cDNA was used as a template for amplification with following primers (5'-3'): CtTF109F: CTT-TTG-GTC-CAC-GAA-GTG-GT; CtTF561R: GCT-TTG-AAG-GAC-AGC-GTT-TC. Control actin primers were: actin1F: ATG-TTT-GAG-ACC-TTC-AAC-TCG-C, actin1R: TAA-CCT-TCR-TAG-ATT-GGG-ACG. PCR conditions were 94°C for 3 min, followed by 30 cycles of: 94°C for 30 sec, 50°C for 30 sec, 72°C for 30 sec, followed by a final extension of 72°C for 10 min. Amplicons were resolved by agarose gel electrophoresis.

### Data mining, sequence analysis and phylogenetic analysis

The complete *Vtg *genes were identified by from the available *An. gambiae, Ae. aegypti *and *Cx. pipiens *genome sequences, and GENBANK sequence data from *Ae. polynesiensis, An. stephensi, An. albimanus *and *Ochlerotatus atropalpus*. Sequence analysis was performed using Blast http://www.ncbi.nlm.nih.gov/blast/ against a non-redundant database. Initial alignments were performed on amino acid sequences using Vector NTI advance 10 (Invitrogen) followed by inference of the nucleotide alignment. Phylogenetic analysis of mosquito *Vtg *genes was conducted on the amino acid alignment using Maximum Likelihood (ML), Bayesian (B) and neighbour-joining (NJ) algorithms. Bayesian phylogenetic analysis was conducted using MrBayes v 3.1.2 [51]. The WAG model of amino acid substitution was selected as the most appropriate using the MCMC sampler to test all fixed rate matrices. The rate variation over sites followed a gamma distribution with a proportion of invariant sites; these parameters were estimated from the data by MrBayes. Analyses were run for 500,000 generations, sampling every 100 generations. The first 25% of generated trees were considered the burnin and discarded. We constructed a 50% majority-rule consensus tree from the remaining trees. Maximum likelihood (ML) phylogenetic analysis was conducted using PHYML [52] with the same parameters estimated for Bayesian analyses. Tree robustness was determined with 100 bootstrap replications. Neighbor-joining analyses were conducted using MEGA v.3.1 [53] with 1000 bootstrap replications.

### Detection of gene conversion events

*Vtg *gene nucleotide alignments were generated as described above. GENECONV was used to detect gene conversion events. Global p-values were calculated based on 1000 permutations of the original data using a BLAST-like search algorithm [28].

### Purifying selection tests and Ps,PN calculation

Analyses were conducted using MEGA v.3.1 [53]. Pairwise dN/dS ratios and significance values were calculated using the Nei-Gojobori method with a Jukes-Cantor correction for multiple hits. Standard errors were calculated by 1000 bootstrap replications.

## Authors' contributions

SC contributed to study design, carried out molecular experiments, conducted bioinformatic analyses and contributed to drafting the manuscript. JSA contributed to molecular analyses. KNPJ assisted with data analysis and contributed to drafting the manuscript. JMS assisted with data analysis. JLR contributed to study design, assisted with data analysis and contributed to drafting the manuscript. All authors read and approved the final manuscript.

## Acknowledgements

This research was funded by NIH/NIAID grant R01AI067371 to JLR.

## Supplementary Material

Additional file 1 Purifying selection of Vitellogenin genes in additional vertebrates and invertebrates. Click here for file

Additional file 2 Gene conversion of Vitellogenin genes in additional vertebrates and invertebrates. Click here for file
